# Metagenomic next-generation sequencing clinches the diagnosis of Legionella pneumonia in a patient with acute myeloid leukemia: A case report and literature review

**DOI:** 10.3389/fcimb.2022.924597

**Published:** 2022-11-21

**Authors:** Siqiang Niu, Limin Zhao

**Affiliations:** ^1^ Zhengzhou University, Zhengzhou, Henan, China; ^2^ Henan Provincial People’s Hospital, Zhengzhou, Henan, China; ^3^ Xinxiang City Central Hospital, Xinxiang, Henan, China; ^4^ Department of Respiratory and Critical Care Medicine, Henan Provincial People’s Hospital, Zhengzhou, Henan, China; ^5^ People's Hospital of Zhengzhou University, Zhengzhou, Henan, China; ^6^ People's Hospital of Henan University, Zhengzhou, Henan, China

**Keywords:** *Legionella pneumonia*, metagenomic next-generation sequencing, literature review, pathogen diagnosis, case

## Abstract

Legionella pneumonia caused by *Legionella pneumophila* is a multi-system disease that is a life-threatening, acute, and severe form of pneumonia. *L. pneumophila* is widespread and the clinical manifestations of Legionella pneumonia are similar to those of typical and atypical pneumonia. Current diagnostic scores and radiologic evidence have limited diagnostic value. Thus, it is likely that many cases of Legionella pneumonia remain unreported. We describe a woman with a medical history of acute myeloid leukemia who suffered from repeated fever, and no relief following initial empirical antibiotic treatment. Ultimately, she was diagnosed with Legionella pneumonia based on metagenomic next-generation sequencing (mNGS). We also performed a systematic review of the literature and identified 5 other patients who were diagnosed with Legionella pneumonia using mNGS, and reviewed their clinical characteristics, biological characteristics, epidemiological features, laboratory results, clinical findings, and treatments. This literature review showed that accurate etiological diagnosis is becoming increasingly essential for a definitive diagnosis and treatment strategies. The clinical manifestations of Legionella pneumonia are non-specific, and many routine laboratory diagnostic tests cannot identify *Legionella.* mNGS, an indispensable approach for identifying microorganisms, can provide a promising tool for the rapid and accurate etiological diagnosis methods contributing to early diagnosis, early treatment, and improved prognosis, especially for uncommon species such as *L. pneumophila.*

## Background

Community-acquired pneumonia (CAP) refers to pulmonary inflammation resulting from infections by bacteria, often *Chlamydia* and *Mycoplasma*, viruses, or other microorganisms. A recent review confirmed that infections by atypical pathogens have increased significantly during recent years, and that *Legionella pneumophila* is increasingly responsible for CAP (Chahin and Opal, 2017).

It is necessary to identify the causative species in CAP so that the most appropriate treatments can be administered. Although a variety of diagnostic methods are commonly used in the clinic, determination of the etiology of CAP remains unknown in approximately 50% of these patients. It is a challenge to achieve a timely diagnosis of Legionella pneumonia because *Legionella* cannot grow on routine bacterial media, and traditional laboratory tests are often time-consuming and expensive.

Moreover, incubation for 3 to 10 days on charcoal-yeast extract agar is required for full confirmation of *Legionella*, further reducing the positive rate of traditional cultures. *Legionella* can be clinically diagnosed by the presence of anti-*Legionella* antibodies. However, most patients only develop anti-*Legionella* antibodies about 3 weeks after the disease onset and the *Legionella* urinary antigen test only detects *L. pneumophila* serogroup 1, leading to delayed diagnosis.

Therefore, accurate and rapid identification of the pathogen responsible for CAP is vital for diagnosis and treatment (Liu et al., 2020). Metagenomic next-generation sequencing (mNGS) is widely applied in clinical laboratories for detecting novel, uncommon, and coinfecting pathogens, and this method provides important diagnostic clues for patients who have repeated fever or are immune-deficient. Here, we report a case who presented with CAP and our use of mNGS of a bronchoalveolar lavage fluid (BALF) sample to identify the causative pathogen as *L. pneumophila.* We then reviewed the literature to identify previous studies, including case reports, case series, and literature reviews, that described patients who had *L. pneumophila* infections with diagnosis by mNGS.

## Main text

A 43-year-old woman with a history of acute myeloid leukemia was admitted to our hospital in July 12, 2020 for repeated fever during the previous 2 months, and a persistent fever for the previous 4 days. Two months previously, she presented with a fever, cough, and expectoration. At that time she was diagnosed with “invasive pulmonary fungal disease” and was given oral voriconazole and biapenem as empirical therapy. Her condition improved gradually after this treatment. Four days prior to the current admission, she was admitted because of a relapse of fever, with a body temperature of 38.6°C.

A physical examination showed that her underarm temperature was

**Figure 1:**
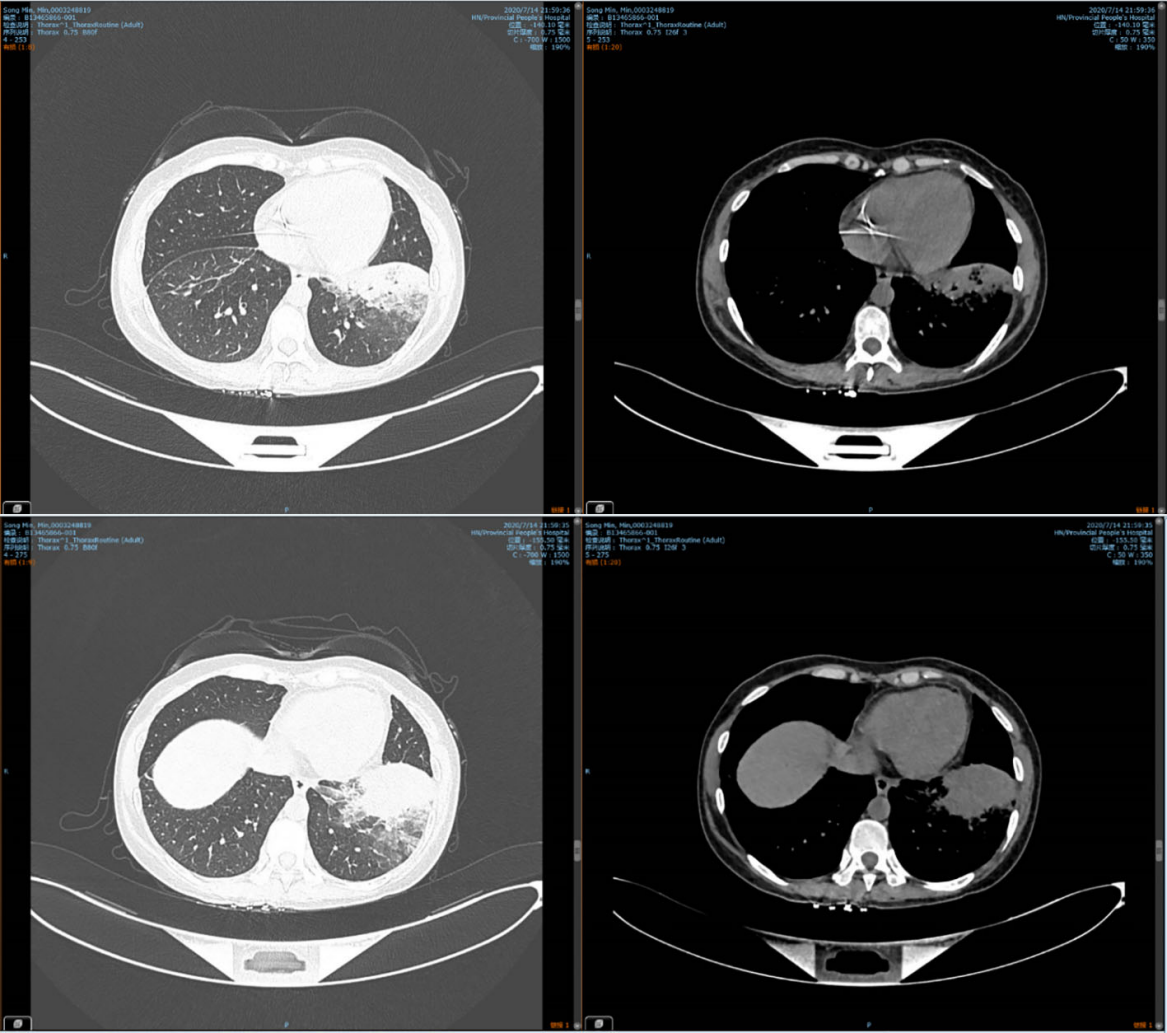
Chest CT on July 14, 2020, showing consolidation in the left lower lung.

38.6°C, pulse was 108 beats/min, breathing was 25 breaths/min, and blood pressure was 109/67 mmHg. There was dullness on lung percussion and strong vocal fremitus with fine crackles in the left lung. Laboratory investigations showed a white blood cell count of 3.61×10^9^ per L (normal: 3.5–9.5), red blood cell count of 3.22×10^12^ per L (normal: 3.8–5.1), hemoglobin concentration of 112g/L(normal: 110-150 g/l), platelet count of 268×10^9^ per L (normal: 125–350), and absolute neutrophil count of 2.02×10^9^ per L (norma: 1.8–6.3). In addition, the 1,3-β-D-glucan level was normal, and an aspergillus antigen test for serum galactomannan was negative. The liver function, renal function, and electrolyte levels were all normal. There were negative results for antinuclear antibody (ANA), extractable nuclear antigen (ENA), antineutrophil cytoplasmic antibodies (ANCA), TB spot test and TB blood culture. Computed tomography (CT) on July 14, 2020 indicated consolidation in the left lower lung **(** **Figure 1** **)**. Therefore, we made a preliminary diagnosis of CAP with acute myeloid leukemia.

As the empirical anti-infective therapy, we administered biapenem (0.3 g, Q6H), but her condition did not improve. On day 3 after admission, the urinary antigen tests for *Streptococcus pneumonia* and anti-*Legionella* antibodies were negative. To identify the etiology, we collected BALF and tested it with mNGS using the MiSeq platform (Illumina). The results indicated 46 sequence readings of *Legionella pneumophila* (**Table 1**) with a coverage of 0.07% (**Table 2**). Sequencing of 16s rRNA sequencing using Nanopore GridION also detected the 17 *L.*pneumophila sequences in the same DNA sample.

**Table 1:** Results of mNGS in the BALF of the patient.

Genus			Species		
Type	Name	Sequence	Name	Sequence	Attention
		Number		Number	
G-	*Legionella*	46	*Legionella pneumophil*	46	high
G-	*Acinetobacter*	19	*Acinetobacter baumannii*	11	low

**Table 2:** *L. pneumophila* coverage from mNGS of the BALF (0.07%).

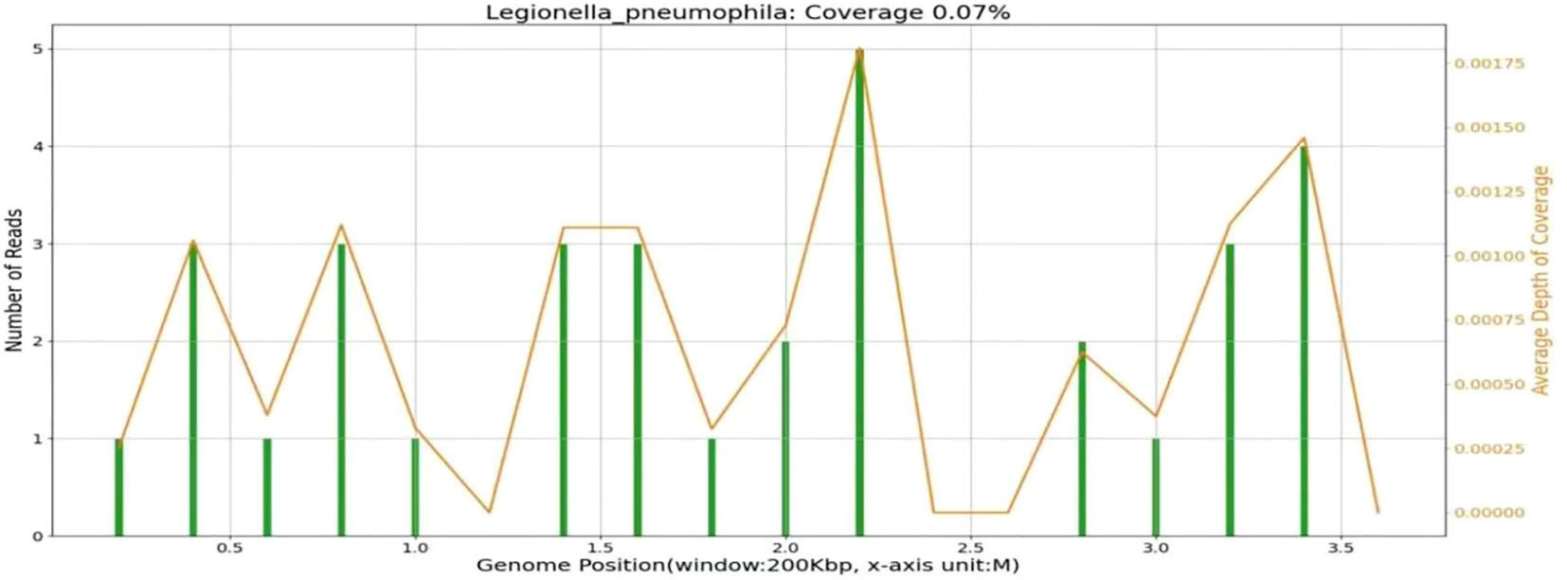

**Figure 2:**
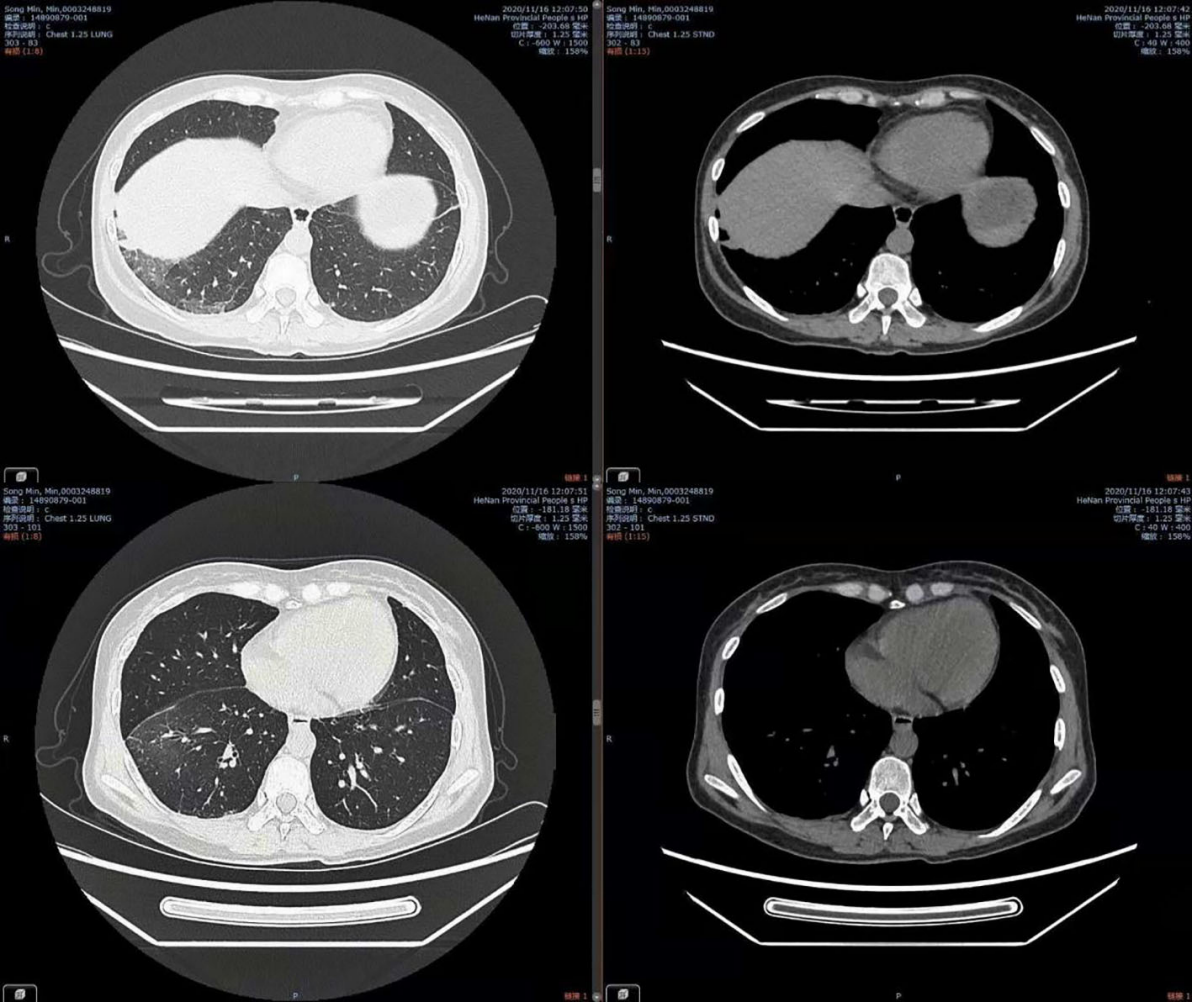
Chest CT on Aug 16, 2020, showing significant absorption of lesions in the left lower lung.

**Figure 3:**
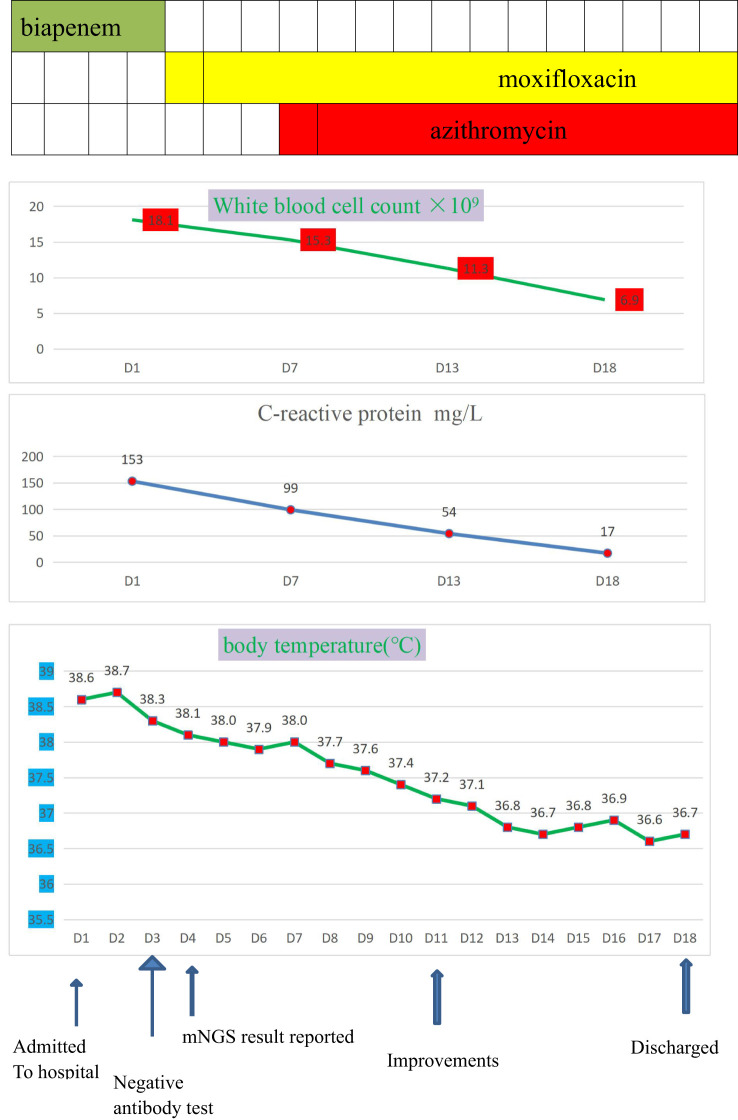
The clinical course of the patient with *Legionella pneumophila* infection.Drug therapies (top), blood indicators (middle), and body temperature (bottom) during hospitalization.

Combined with her medical history, clinical symptoms, physical signs, results from auxiliary examinations, and mNGS, we diagnosed the patient with Legionella pneumonia and adjusted the treatment plan to intravenous moxifloxacin and azithromycin. After 14 days of treatment in the hospital, the patient’s fever had resolved, and she was discharged, and prescribed antimicrobial agents as sequential oral therapy. A CT follow-up after one month (August 16, 2020) indicated the pulmonary lesions in the left lower lung were significantly absorbed (**Figure 2**). Records of her white blood cell count, C-reactive protein, body temperature, and other results indicated gradual improvements during her hospital stay (**Figure 3**).

## Discussion

*Legionella* is an aerobic Gram-negative bacterium that is 0.5-1 μm wide and 2-50 μm long. They can be grown on complex media (buffered charcoal-yeast extract agar with α-ketoglutarate) at a pH of 6.4-7.2 and a temperature of 35°C (Viasus et al., 2019). In 1976, an outbreak of acute febrile respiratory disease in Philadelphia (U.S.) among members of the American Legion led to many deaths. The causative bacterial species was subsequently identified and named *Legionella pneumophila*. A retrospective study reported evidence of species in this genus from clinical samples that were collected in 1943. There are now more than 50 known species of *Legionella*, and at least 20 of them are pathogens that can cause pneumonia. The most common species are *L. pneumophila* (85–90%), followed by *L. micdadei* (5%–10%), *L. bozemanii* and *L. dumofi.* These different species have similar morphologies and biochemical characteristics, and cause diseases with similar symptoms (Garcia-Vidal and Carratalà, 2006).

*L. Pneumophila* is widespread in natural freshwater, and many of the other species in this genus are ubiquitous in warm water, ideally with temperatures ranging from 20 to 50°C. This temperature can change the balance of protozoa and bacteria and lead to the rapid multiplication of *Legionella*. There is evidence indicating that *Legionella* grows within amoebas, which then transfer the *Legionella* from drinking water to susceptible populations (Bell et al., 2021). *L. pneumophila* has optimal growth in warm water in the presence of amoebae and freshwater bacteria, and it proliferates as much within amoebas as in pulmonary macrophages in the lung. Water supply systems and cooling towers create aerosols, and when they become contaminated, a susceptible host may become infected with *Legionella* by inhalation. Because aerosol droplets smaller than 10 µm can enter deep into the lungs, they were once considered the primary mode of *Legionella* transmission. However, aspiration is now considered the more common mode of transmission. Thus, pathogenic factors, including the presence of bacteria in an aquatic environments, unique combinations of nutrients, rapid multiplication of bacteria, and transmission to susceptible populations *via* aerosol, may increase the risk of infection.

Although Legionella pneumonia can be life-threatening, infections can be overlooked because many clinical laboratories lack the capacity to isolate and identify *Legionella*. Several interventions should be used to prevent this disease. These include increasing the temperature of possibly contaminated water beyond the growth range of *Legionella*, controlling the growth of *Legionella* biofilms, and routine testing of water samples from cooling towers. Identifying *Legionella* as a causative agent of an infection is an essential public health measure, so appropriate clinical surveillance and routine diagnostic testing, such as culturing and urine antigen testing(UAT), should be performed in high-risk patients. Finally, the use of monochloramine rather than chlorine as a biocide in municipal water systems is more effective in preventing *Legionella* transmission. Despite the increased implementation of sufficient measures for monitoring and controlling *Legionella* during recent years, the definitive origins of most sporadic infections remain unknown.

A patient infected with *Legionella* may develop Legionnaires’ disease (Legionella pneumonia) or Pontiac fever (a less severe non-pneumonia disease). Legionella pneumonia is a multi-system disease that is associated with high mortality, often involves the lungs and gastrointestinal tract, and the initial symptoms are fever, cough, headache, and myalgias, followed by a rapidly rising fever. Patients may also experience chills, pleuritic chest pain, confusion, shortness of breath, excess sputum production, anorexia, nausea, and diarrhea, although extra-pulmonary infections such as skin and soft-tissue infections, bacteremia, endocarditis, and septic arthritis are rare (Escoll et al., 2013). Patchy or interstitial infiltration of the lungs with progression towards nodular consolidation is often evident in chest CT scans. Most patients develop progressive illness and toxication over the first 3 to 6 days, and death may occur due to tension pneumothorax, shock, respiratory failure, or multiple organ dysfunction syndrome (MODS). The overall mortality rate ranges from 3% to 33%, with higher rates in children and in adults who have chronic lung disease, hematologic malignancies, end-stage renal disease, and immunosuppression.

Pontiac fever is a mild non-pneumonia disease characterized by mild fever, chills, dry cough, and myalgia, and an incubation of 6 h to 5 days. These patients do not require treatment with antibiotic therapy, and many remain undiagnosed. The pathogenesis of Pontiac fever is not well known, but it may not be directly attributable to *Legionella* infection. Instead, it may be a reaction to an endotoxin or hypersensitivity to components of *Legionella* or *Legionella*-carrying amoebae.

It is often difficult for clinicians to distinguish Legionella pneumonia from other causes of pneumonia because there are no specific clinical manifestations. It is vital to develop an accurate diagnostic scoring system because some specialists questioned the definition of Legionella pneumonia based on a single laboratory test. There are currently several diagnostic tests for Legionella pneumonia, such as culture, UAT, direct fluorescent antibody (DFA) testing, and *Legionella*-specific polymerase chain reaction (PCR). Culture diagnosis was previously the gold standard for diagnosis, but is rarely used at present because it is very time-consuming. UAT is widely performed in clinical practice, but it is only sensitive to *Legionella pneumophila* serogroup 1 (Pierre et al., 2017). The additional limitations of the UAT are that immediate testing of specimens is necessary because the antigen degrades over time and that it has low sensitivity and is therefore less useful for patients with mild infections (Cunha, 2006). DFA testing is commonly performed, but this test cannot detect all clinical species of *Legionella*.

Despite the rapid development of molecular tools, such as *Legionella*-specific PCR, they are rarely used in clinical practice, A recent European study reported that only 2% of the 11,832 probable or confirmed cases of Legionella pneumonia were identified by PCR. Moreover, this technique is not yet well established in some areas of China. Although PCR has greater diagnostic value than the UAT, the results of culture and PCR are often discrepant due to the protection of *Legionella pneumophila* within protozoa-like amoebae that can’t grow on culture media.

Clinical data showed that *Legionella* was susceptible to clarithromycin, azithromycin, levofloxacin, and erythromycin (Michael Dunne et al., 2017). There is also evidence supporting the safety and effectiveness of sequential therapy with levofloxacin and macrolides, and rifampin(10–20mg/kg/day) may be used in a patient who has a delayed response to treatment. The mortality rate is higher if a patient is untreated or if there is a delay in the administration of an appropriate antibiotic. Patients with nosocomial infections, and those with diabetes, immunosuppression, and malignancies have an increased risk of mortality (Griffin et al., 2010).

mNGS is increasingly applied for unbiased culture-independent identification of pathogens. Since its introduction, mNGS has enhanced existing gene sequencing platforms because it allows the sequencing of thousands to millions of DNA molecules in a single machine. It can perform well in identifying rare, novel, difficult-to-detect, and coinfected pathogens directly from clinical samples and present great potential for detection of pathogenic bacteria, contributing to the provision of new diagnostic evidence that can be used to guide treatment options and improve antimicrobial drug applications (Hilbi et al., 2010). The limitations of mNGS are the high costs, analytical sensitivity, complex laboratory workflow, and susceptibility to contamination.

Our patient suffered from repeated fever within 2 months due to a pulmonary infection, and we used mNGS to identify *Legionella pneumophila* as the causative pathogen. After targeted clinical adjustment, the patient’s condition improved and she was discharged after 18 days. We believe that clinicians should be alert to rare and complex pathogens in patients who have leukemia, tumours, HIV infection, diabetes or received organ transplantation, all of which lead to immunosuppression. Thus, if a pathogen cannot be identified by conventional detection methods, mNGS should be considered so there can be an appropriate adjustment of medication (Han et al., 2019). Although mNGS confronts several dilemmas and hurdles, many physicians believe these limitations can be defeated by updated technologies and recognize mNGS as an effective way to resolve clinical infection problems. With the development of lower cost, an easier workflow and simplified interpretation criteria, mNGS will be widely used in clinical practice (Li et al., 2021).

We also performed a literature search of PubMed to identify other clinical studies that described patients diagnosed with *L. pneumophila* infection based on mNGS using the keywords “*Legionella pneumophila* Infection” and “Next-Generation Sequencing”. We identified at least 5 previous cases of Legionella infection diagnosed using mNGS, most of whom were immunocompromised due to a condition such as cancer, stem cell transplantation, systemic lupus erythematosus (SLE), and smoking history **(** **Tables 3**, **4** **). **The initial symptoms of these patients were fever, vomiting, diarrhea, kidney dysfunction, chest pain, and dyspnea. Most patients had negative results for *L. pneumophila* based on serum anti-*Legionella* antibodies, urine antigens, and sputum cultures. To identify the sources of infection, these studies used mNGS to analyze BALF, blood, sputum, pleural effusion, and purulent aspirates from arthrocentesis.

**Table 3:** Clinical data of 5 patients diagnosed with *legionella* infections based on mNGS; UCBT, umbilical cord blood stem cell transplantation.

No.	Year	Sex	References	Initial symptoms	Underlying diseases
1	65	Female	Huahua Yi	Fever, Gastroenteritis	Breast cancer
2	46	Male	Ruiming Yue	Fever, Chills, Diarrhea	Smoking history
3	7	Female	Yangyan Wang	High fever,	UCBT
4	53	Male	Cheng Lei	Fever, Dyspnea	Smoking history
5	54	Male	Yingnan Huang	Progressive joint pain	Oral corticosteroid

**Table 4:** Type of sample, species, therapy, confirmation method, and outcome of 5 patients previously diagnosed with *Legionella* infections based on mNGS.

No.	Sample	Species	Therapy	Confirmed method	Outcome
1	Blood, Sputum, pleural effusion	*L. pneumophila*	MSF	PCR	Recovery
2	Blood, BALF	*L. pneumophila*	ECMO, CRRT, MSF, AZF	None	Progression
3	Blood	*L. pneumophila*	AZF, CFP	TOFMS	Recovery
4	Blood, BALF	*L.gormanii*	MSF, MEC	CE&Sanger sequencing	Recovery
5	Purulent aspirate	*L. micdadei*	LEV	Gene sequencing	Recovery

MEC, meropenem; MSF, moxifloxacin; LEV, levofloxacin; AZF, azithromycin; CFP, cefoperazone; TOFMS, time-of-flight mass spectrometry; CE, capillary electrophoresis.

Patient No. 1 was a 65-year-old female with a history of breast cancer who was admitted to the Emergency Room because of vomiting and symptoms of severe respiratory infection. mNGS assays of sputum, blood and pleural effusion indicated infection by *L. pneumophila*, and this was confirmed by a PCR assay of the pleural infusion and sputum (Yi et al., 2020).

Patient No. 2 was a 46-year-old male with a long history of smoking. He suffered from acute onset of CAP and rapidly progressed to ARDS and kidney damage. A mNGS assay of his BALF indicated *L. pneumophila* and *Klebsiella pneumonia*. This patient was given venovenous extracorporeal membrane oxygenation (VV-ECMO) combined with continuous renal replacement therapy (CRRT) in the intensive care unit. His condition progressed rapidly, showing a brief improvement before manifestations of dyspnea, chills, and fever again (Yue et al., 2021).

Patient No. 3 was a 7-year-old female who received umbilical cord blood stem cell transplantation (UCBT) due to type 2 myelodysplastic syndrome, and subsequently developed a high fever later. A mNGS assay of her BALF indicated *L. pneumophila* infection, and this was confirmed by time-of-flight mass spectrometry (TOFMS) on selective MWY medium. The patient recovered after treatment with moxifloxacin and azithromycin (Wang et al., 2021).

Patient No. 4 was a 53-year-old male with a history of smoking who presented with clinical symptoms of cough, chest pain, fever, and dyspnea. A mNGS assay of his BALF revealed *L. gormanii* infection, and this was confirmed by capillary electrophoresis (CE) and Sanger sequencing (Lei et al., 2022). After adjusting the antibiotics to meropenem and moxifloxacin, she achieved a good recovery.

Patient No. 5 was a 54-year-old male who had a history of using oral corticosteroids for SLE. He presented with a recent swelling and progressive pain in the right metacarpophalangeal joint (MCP). A mNGS assay of purulent aspirate obtained by arthrocentesis of the affected joint indicated abundant *L. micdadei*, which was confirmed by 16s rRNA gene sequence analysis. The patient was given oral levofloxacin for 60 days, and had an almost complete recovery (Huang et al., 2019). Among these 5 patients, there were 3 infections by *L. pneumophila*, 1 by infection *L. gormanii*, and 1 infection by *L. micdadei.*

## Conclusion

Legionella pneumonia has non-specific symptoms. The traditional methods used for identification of *Legionella* from clinical samples have significant limitations. mNGS is a fast and effective methodology for identification of pathogens from clinical samples and is especially useful for identification of uncommon species, such as *Legionella pneumophila*.

## Data availability statement

The datasets for this article are not publicly available due to concerns regarding participant/patient anonymity. Requests to access the datasets should be directed to the corresponding author.

## Ethics statement

Written informed consent was obtained from the individual(s) for the publication of any potentially identifiable images or data included in this article.

## Author contributions

SN wrote the first draft of the manuscript and participated in the literature collection and evaluation. LZ supervised and critically revised the manuscript. All authors contributed to the article and approved the submitted version. Written consent for publication was obtained from the patient.

## Conflict of interest

The handling editor LA and the reviewer XG declared a shared parent affiliation with the authors at the time of review.

## Publisher’s note

All claims expressed in this article are solely those of the authors and do not necessarily represent those of their affiliated organizations, or those of the publisher, the editors and the reviewers. Any product that may be evaluated in this article, or claim that may be made by its manufacturer, is not guaranteed or endorsed by the publisher.

## References

[B1] Bell H. Chintalapati S. Patel P. Halim A. Kithas A. Sarah A.. (2021). Legionella longbeachae pneumonia: Case report and review of reported cases in non-endemic countriess. IDCases 23, e01050. doi: 10.1016/j.idcr.2021.e01050.7 33511033 PMC7817369

[B2] Chahin A. Opal S. M. (2017). Severe pneumonia caused by legionella pneumophila: Differential diagnosis and therapeutic considerations,a review. Infect. Dis. Clin. North Am. 31 (1), 111–121. doi: 10.1016/j.idc.2016.10.009.1 28159171 PMC7135102

[B3] Cunha B. A. (2006). The atypical pneumonias: clinical diagnosis and importance, a review. Clin. Microbiol. Infect. 12 Suppl 3, 12–24. doi: 10.1111/j.1469-0691.2006.01393.x 16669925 PMC7128183

[B4] Escoll P. Rolando M. Gomez-Valero L. Buchrieser C.. (2013). From amoeba to macrophages: exploring the molecular mechanisms of legionella pneumophila infection in both hosts. Curr. Top. Microbiol. Immunol. 376, 1–34. doi: 10.1007/82_2013_351 23949285

[B5] Garcia-Vidal C. Carratalà J. (2006). Current clinical management of legionnaires' disease. Expert Rev. Anti Infect. Ther. 4 (6), 995–1004. doi: 10.1586/14787210.4.6.995 17181416

[B6] Griffin A. T. Peyrani P. Wiemken T. Arnold F.. (2010). Macrolides versus quinolones in legionella pneumonia: results from the community-acquired pneumonia organization international study. Int. J. Tuberc Lung Dis. 14 (4), 495–499. 20202309

[B7] Han D. Li Z. Li R. Tan P. Zhang R. Li J.. (2019). mNGS in clinical microbiology laboratories: on the road to maturity. Crit. Rev. Microbiol. 45(5–456), 668–685. doi: 10.1080/1040841X.2019.1681933 31691607

[B8] Hilbi H. Jarraud S. Hartland E.. (2010). Update on legionnaires' disease: pathogenesis, epidemiology, detection and control. Mol. Microbiol. 76 (1), 1–11. doi: 10.1111/j.1365-2958.2010.07086.x 20149105 PMC2914503

[B9] Huang Y. Ma Y. Miao Q. Pan J. Hu B. Gong Y.. (2019). Arthritis caused by legionella micdadei and staphylococcus aureus: metagenomic next-generation sequencing provides a rapid and accurate access to diagnosis and surveillance. Ann. Trans. Med. 7 (20), 589. doi: 10.21037/atm.2019.09.81 PMC6861802 31807570

[B10] Lei C. Zhou X. Ding S. Xu Y. Yang B. Gou W.. (2022). Case report: Community-acquired legionella gormanii pneumonia in an immunocompetent patient detected by metagenomic next-generation sequencing. Front. Med. 9. doi: 10.3389/fmed.2022.819425 PMC8831763 35155502

[B11] Li N. Cai Q. Miao Q. Song Z. Fang Y. Hu B.. (2021). High-throughput metagenomic for identification of pathogens in the clinical settings. Small Methods 5 (1), 2000792. doi: 10.1002/smtd.202000792 33614906 PMC7883231

[B12] Liu D. Zhang C. Wang Y. Xu S.. (2020). Challenges and considerations on quality control and evaluation of pathogen metagenomic next-generation sequencing. Sheng Wu Gong Cheng Xue Bao. 36 (12), 2598–2609. doi: 10.13345/j.cjb.200377 33398957

[B13] Michael Dunne W. Jr Picot N. van Belkum A. (2017). Laboratory tests for legionnaire's disease. Infect. Dis. Clin. North Am. 31 (1), 167–178. doi: 10.1016/j.idc.2016.10.012 27979684

[B14] Pierre D. M. Baron J. Yu V. L. Stout J.E.. (2017). Diagnostic testing for legionnaires' disease. Annals of Clinical Microbiology and Antimicrobials 16, 1, 59. doi: 10.1186/s12941-017-0229-6 28851372 PMC5576257

[B15] Viasus D. Calatayud L. McBrown M. V. Ardanuy C. Carratalà J.. (2019). Urinary antigen testing in community-acquired pneumonia in adults: An update, a review. Expert Rev. Anti Infect. Ther. 17 (2), 107–115. doi: 10.1080/14787210.2019.1565994 30618315

[B16] Wang Y. Dai Y. Lu H. Chang W. Ma F. Wang Z.. (2021). Case report: Metagenomic next-generation sequencing in diagnosis of legionella pneumophila pneumonia in a patient after umbilical cord blood stem cell transplantation. Front. Med. 8. doi: 10.3389/fmed.2021.643473 PMC8232522 34179036

[B17] Yi H. Fang J. Huang J. Liu B. Qu J. Zhou M.. (2020). *Legionella pneumophila* as cause of severe community-acquired pneumonia, China. Emerging Infect. diseases. 26 (1), 160–162. doi: 10.3201/eid2601.190655 PMC6924908 31855541

[B18] Yue R. Wu X. Li T. Chang L. Huang X. Pan L.. (2021). Early detection of *Legionella pneumophila* and *Aspergillus* by mNGS in a critically ill patient with legionella pneumonia after extracorporeal membrane oxygenation treatment: Case report and literature review. Front. Med. 8. doi: 10.3389/fmed.2021.686512 PMC8277993 34277662

